# Successful Isolation of Diverse *Verrucomicrobiota* Strains through the Duckweed-Microbes Co-cultivation Method

**DOI:** 10.1264/jsme2.ME24019

**Published:** 2024-09-12

**Authors:** Yasuhiro Tanaka, Erina Tozawa, Tomoki Iwashita, Yosuke Morishita, Hideyuki Tamaki, Tadashi Toyama, Masaaki Morikawa, Yoichi Kamagata, Kazuhiro Mori

**Affiliations:** 1 Graduate School of Life and Environmental Sciences, University of Yamanashi, 4–4–37 Takeda, Kofu, Yamanashi 400–8510, Japan; 2 Graduate School of Engineering, University of Yamanashi, 4–3–11 Takeda, Kofu, Yamanashi 400–8511, Japan; 3 Bioproduction Research Institute, AIST, 1–1–1 Higashi, Tsukuba, Ibaraki 305–8566, Japan; 4 Division of Biosphere Science, Graduate School of Environmental Science, Hokkaido University, Kita–10 Nishi–5, Kita–ku, Sapporo 060–0810, Japan

**Keywords:** *Verrucomicrobiota*, duckweed, aquatic plant, microbial isolation

## Abstract

The “duckweed-microbes co-cultivation method” is a microbial isolation technique that effectively recovers diverse microbes, including rarely cultivated bacterial phyla, from environmental samples. In this method, aseptic duckweed and microbes collected from an environmental sample are co-cultivated for several days, and duckweed-associated microbes are then isolated from its roots using a conventional agar plate-based cultivation method. We herein propose several improvements to the method in order to specifically obtain members of the rarely cultivated bacterial phylum, *Verrucomicrobiota*. In systems using river water as the inoculum, the marked enrichment of *Verrucomicrobiota* was observed after 10 days of co-cultivation, particularly in the roots and co-cultivated media. We also successfully isolated 44 strains belonging to subdivisions 1, 3, and 4 of the phylum *Verrucomicrobiota* from these systems. This was achieved by changing the concentration of nitrogen in the co-cultivation medium, which is known to affect duckweed growth and/or metabolism, and by subjecting the fronds and co-cultivated media as well as the roots after co-cultivation to microbial isolation.

Recent advances in mole­cular-based ana­lysis techniques have estimated the existence of 2.2–4.3 million species of diverse prokaryotes in nature (Louca *et al.*, 2019). However, most are yet-to-be cultured or uncultivable, and only approximately 23,500 species have been isolated, cultured, and validly published as of the writing of this manuscript (List of Prokaryotic names with Standing in Nomenclature; https://www.bacterio.net/). Although these species are distributed over 43 phyla, more than 90% belong to the bacterial phyla *Pseudomonadota*, *Actinomycetota*, *Bacillota*, and *Bacteroidota*. Therefore, many archaeal and bacterial species within other phyla are regarded as “rarely cultivated microbes”. In addition, mole­cular-based ana­lyses targeting 16S rRNA gene sequences estimated the presence of more than 70 candidate phyla or divisions within bacterial and archaeal domains, which are called “microbial dark matter” (Rinke *et al.*, 2013; Lok, 2015; Parks *et al.*, 2018; Zha *et al.*, 2022). On the other hand, since numerous novel and useful functional genes have been detected based on metagenomic ana­lyses of various samples, so-called “rarely cultivated microbes” and “microbial dark matter” are expected to become new biological resources (Michalska *et al.*, 2015; Danso *et al.*, 2019; Verma *et al.*, 2021; Sysoev *et al.*, 2021; Zha *et al.*, 2022).

In our previous studies, a wide variety of microbes, including rarely cultivated bacterial groups, such as the phyla *Verrucomicrobiota*, *Acidobacteriota*, and *Armatimonadota*, were easily isolated from the roots of aquatic macrophytes using a conventional method (Matsuzawa *et al.*, 2010; Tanaka *et al.*, 2012; Tanaka *et al.*, 2017). Based on the findings obtained, we developed a new microbial isolation method called the “duckweed-microbes co-cultivation method”, in which aseptic duckweed is inoculated with microbes from environmental samples and co-cultivated for a specific period, and the resultant roots of the plant are then used as a source for microbial isolation (Tanaka *et al.*, 2018). We successfully isolated various microbes by employing this method, including members of the phyla *Verrucomicrobiota* and *Armatimonadota* that had not been previously isolated from the original sample (homogenates of Japanese loosestrife root) (Tanaka *et al.*, 2018). Although the reason why this method effectively isolated these microbes has not yet been elucidated in detail, it may be attributed to the reconstruction of the microbial community on the roots of duckweed through interactions between microbes and duckweed involving physical and chemical factors (exudates), in which the predominance of fastidious microbes and/or the conversion to a culturable state may play a role.

Terrestrial plants are known to change the composition of root exudates in response to chemical, physical, and biological factors, which, in turn, affects microbial communities in the rhizosphere (Yang and Crowley, 2000; Marschner *et al.*, 2002; Das *et al.*, 2017; Pascale *et al.*, 2020; Pantigoso *et al.*, 2022). These findings indicate that changes in the factors that have an impact on exudate secretion by duckweed modify the microbial community on plants, consequently enhancing the diversity of microbial species obtained through the “duckweed-microbes co-cultivation method”. In addition, if the factor directly affects the growth and distribution of microbes, synergistic effects may be expected. On the other hand, we recently reported that the microbial communities that formed on the fronds, a leaf-like structure, of duckweed differed from those on the roots. Therefore, the fronds have emerged as an alternative source for isolating rare and novel microbes in addition to the roots (Iwashita *et al.*, 2020). In the present study, with the aim of obtaining data to support these speculations, we co-cultivated duckweed and microbes in media with varying concentrations of nitrate, a factor known to affect plant growth, metabolism (Petersen *et al.*, 2021), and microbial composition in environmental samples (Haukka *et al.*, 2006; Pei *et al.*, 2021; Xiao *et al.*, 2023; Zhang *et al.*, 2023). We then compared the microbial community structures that formed in the roots, fronds, and co-cultivated medium based on 16S rRNA gene amplicon sequencing.

The phylum *Verrucomicrobiota* is a phylogenetically diverse phylum that is ubiquitously distributed in nature. However, only 69 species have so far been validly published due to their difficulty to cultivate as stated above (List of Prokaryotic names with Standing in Nomenclature; https://www.bacterio.net/). Recent metagenomic ana­lyses suggested the importance of this phylum in terrestrial ecological processes, *i.e.*, carbon, sulfur, and nitrogen cycles (Kalam *et al.*, 2022). Additionally, the presence of useful features, such as plant growth promotion and second metabolite(s) biosynthesis, have been predicted (Kalam *et al.*, 2022). On the other hand, the microbes within this phylum were previously isolated from both the roots and fronds of duckweed, and their abundance in aquatic environmental samples is considered to be affected by the concentration of nitrate (Haukka *et al.*, 2006; Pei *et al.*, 2021). Therefore, we also investigated which samples in the duckweed-microbes co-cultivation system have potential as a source for isolation, and whether their abundance and isolation yields differed under varying nitrate concentrations in the system.

## Materials and Methods

### Plant material

We used aseptic duckweed (*Spirodela polyrhiza*), which was prepared with the method described in a previous study (Toyama *et al.*, 2006). This plant was grown in medium comprising 36.1‍ ‍mg KNO_3_, 293‍ ‍mg K_2_SO_4_, 3.87‍ ‍mg NaH_2_PO_4_, 103‍ ‍mg MgSO_4_·7H_2_O, 147‍ ‍mg CaCl_2_·2H_2_O, 3.33‍ ‍mg FeSO_4_·7H_2_O, 0.95‍ ‍mg H_3_BO_3_, 0.39‍ ‍mg MnCl_2_·4H_2_O, 0.03‍ ‍mg CuSO_4_·5H_2_O, 0.08‍ ‍mg ZnSO_4_·7H_2_O, and 0.254‍ ‍mg H_2_MoO_4_·H_2_O in 1 L of distilled water (pH 7.0) reported by Toyama *et al.* (2006) (hereafter named Toyama medium).

### Duckweed-microbes co-cultivation

**Fig. 1.**
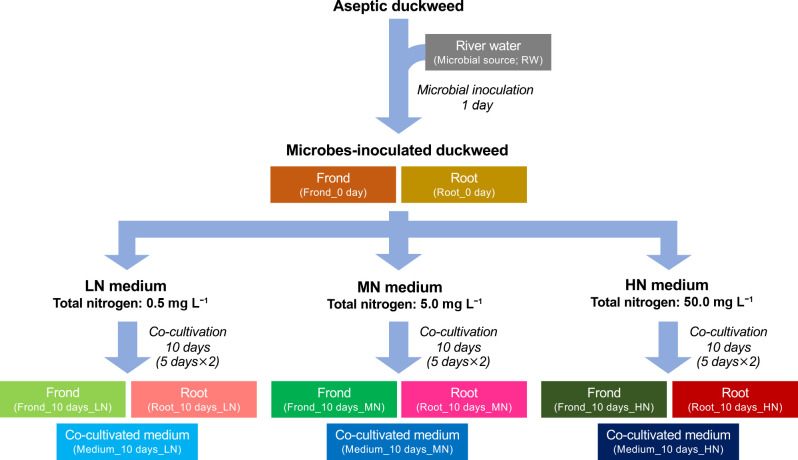
Schematic image for constructing the “duckweed-microbes co-cultivation system” and sample information used in this study. Sample IDs are shown in parentheses.

A schematic image of the duckweed-microbes co-cultivation and samples targeted for the microbial ana­lysis and isolation are summarized in Fig. 1 (more detailed information may be found in Fig. S1).

River water collected from Aikawa river located within Kofu city (Kofu, Yamanashi, Japan; 35°40′36.8″N 138°33′45.7″E) in July, 2017 was used as the microbial source. Thirty-three plants of aseptic duckweed were transplanted into 200‍ ‍mL of the river water sample in a 500-mL plant culture bottle to inoculate microbes, and were then kept in a plant growth chamber (PPFD of 132‍ ‍μmol m^–2^ s^–1^; 16:8‍ ‍h light-dark cycle) at 25°C. After a 1-day incubation, plants were gently washed twice with 90‍ ‍mL of sterilized Toyama medium. Three washed duckweed plants were used for total DNA extraction (see below). Residual microbe-inoculated duckweeds (30 plants) were divided into 3 groups, and the plants in each group (10 plants) were transferred into three different media (100‍ ‍mL in a 500-mL plant culture bottle), hereinafter referred to as LN, MN, and HN media, which included the same constituents as Toyama medium, except for the concentration of KNO_3_; LN, MN, and HN media included 0.5, 5.0, and 50.0‍ ‍mg of total nitrogen (T-N) L^–1^, respectively. Culture bottles including the duckweeds were incubated in a plant growth chamber (PPFD of 132‍ ‍μmol m^–2^ s^–1^; 16:8‍ ‍h light-dark cycle) at 25°C. After 5 days (1st batch), 10 plants were collected from each medium, transferred to fresh medium, and cultivated for 5 days (2nd batch) in the plant growth chamber (total 10 days of cultivation).

### Microbial community ana­lysis

Total nucleic acid extraction from duckweed and water samples was performed as described in our previous study (Iwashita *et al.*, 2020). Briefly, 3 duckweed plants were washed twice with sterilized fresh medium that was used for their cultivation (LN, MN, or HN medium) and then separated into fronds and roots by cutting with a sterilized scalpel. The resultant plant samples were subjected to DNA extraction using Cica Geneus DNA Extraction Reagent (Kanto Chemical). Fifty milliliters of a river water sample or medium sample after the duckweed-microbes co-cultivation (hereafter referred to as “co-cultivated medium”) was filtrated through a membrane filter (Isopore membrane filters, pore size of 0.22‍ ‍μm; Merck). Microbes trapped on the filter were suspended with 500‍ ‍μL of TE buffer, and an aliquot (100‍ ‍μL) of this suspension was applied to DNA extraction using Cica Geneus DNA Extraction Reagent.

To identify the microbial communities in samples, 16S rRNA amplicons targeting the V4 region were analyzed using a next generation sequencing (NGS)-based technique. Extracted DNAs were applied to the first PCR using the primer set, Eub-515F (5′-ACACTCTTTCCCTACACGACGCTCTTCCGATCTGTGCCAGCMGCCGCGGTAA-3′; the sequence for the 2nd PCR was underlined) and Eub-806R (5′-GTGACTGGAGTTCAGACGTGTGCTCTTCCGATCTGGACTACHVGGGTWTCTAAT-3′; the sequence for the 2nd PCR was underlined) as previously described (Ghaju Shrestha *et al.*, 2017). The second PCR amplification and sequencing using the MiSeq sequencer (Illumina) were performed by FASMAC. Non-chimeric sequences were analyzed by 16S rRNA gene-based microbiome taxonomic profiling (MTP) of EzBioCloud (https://www.ezbiocloud.net/contents/16smtp), which were classified into the phylum or genus level. During this operation, chloroplast and mitochondrial operational taxonomic units (OTUs) were discarded.

A heat map was created by R (version 4.2.3) using the gplots package (3.1.3), and a cluster ana­lysis was also performed by the dist function “Canberra” and the group average method. A principal coordinate ana­lysis (PCoA) was conducted with the Jensen-Shannon metric on the MTP set browser of EzBioCloud.

### Microbial isolation and screening of *Verrucomicrobia*

The isolation of microbes from duckweed and water samples was conducted by the method using low-nutrient DTS agar plates reported in our previous study (Iwashita *et al.*, 2020). Briefly, as described in the section on the mole­cular-based microbial community ana­lysis, 3 duckweed plants were separated into fronds and roots after being washed twice with sterilized medium. The parts were homogenized in 10‍ ‍mL of sterilized DTS medium using the Vibra-Cell Ultrasonic Liquid Processor VCX 130 (130 W, 20 kHz; Sonics) for 2‍ ‍min (fronds) or 1‍ ‍min (roots). Homogenates and water samples (river water and co-cultivated media) were subjected to microbial cultivation after serial dilutions. After 14 days of cultivation at 25°C, the colonies that emerged on the plate were randomly selected and subcultured on new DTS agar plates. To screen microbes belonging to the phylum *Verrucomicrobia* from subcultured strains, a colony direct PCR technique using the degenerated primer set, VMB537f (5′-GCCAGCAGCCGCGGTAATACA-3′) and VMB1295r (5′-GCAGMCTNCAATCTGAACTGRGC-3′) (O’Farrell and Janssen, 1999), which specifically detects the 16S rRNA gene sequences of this phylum, and SapphireAmp fast PCR Master mix (TaKaRa Bio) was employed. The amplification program for the reaction was as follows: an initial activation step at 94°C for 60‍ ‍s, followed by 35 cycles of denaturation at 98°C for 5‍ ‍s, annealing at 60°C for 5‍ ‍s, and extension at 72°C for 10‍ ‍s, and a final extension step of 72°C for 15 s. The strains that passed the above screening were applied to the PCR amplification of almost the full length of the 16S rRNA gene using the bacterial universal primer set, EUB8F (5′-AGAGTTTGATCMTGGCTCAG-3′: corresponding to positions 8–27 of the *Escherichia coli* 16S rRNA gene) (Weisburg *et al.*, 1991) and EUB1512R (5′-ACGGYTACCTTGTTACGACTT-3′; corresponding to positions 1492–1512 of the *E. coli* 16S rRNA gene) (Kane *et al.*, 1993). Reactions were conducted as previously described (Matsuzawa *et al.*, 2010), and PCR products from the strains were purified with the GFX PCR DNA and Gel purification kit (Merck) and sequenced with the primer EUB907R (Tamaki *et al.*, 2005) or EUB1512R (only for strain AR-M50MN). Sequences were compared with those present in the public database using the BLAST program (https://blast.ncbi.nlm.nih.gov/Blast.cgi) to check their taxonomic accuracy.

### Sequencing data

The 16S rRNA amplicon sequences obtained by the NGS-based technique were deposited in the DNA Data Bank of Japan under the accession number DRA017435. GenBank/EMBL/DDBJ accession numbers for the 16S rRNA gene sequences of *Verrucomicrobia* isolates are LC785828–LC785871.

## Results and Discussion

### Microbial communities in duckweed-microbes co-cultivation systems

A 16S rRNA gene amplicon sequencing ana­lysis was conducted to identify the microbial community distributed in the fronds, roots, and co-cultivated medium of three duckweed-microbes co-cultivation systems with different nitrate concentrations. In 12 samples including river water (the original microbial source), between 4,449 and 97,209 of archaeal or bacterial 16S rRNA gene sequences were obtained and grouped into 343 to 7,734 OTUs, with Good’s coverage of the library ranging between 97.89 and 99.92% (Table S1). The microbial evenness of duckweed-microbes co-cultivation-related samples based on the Shannon index showed no marked differences among the samples. In contrast, Chao 1 scores showed that the richness of microbes was slightly higher in the roots than in the fronds and co-cultivated media (the scores for fronds were equivalent to those for co-cultivated media) (Table S1). Shannon and Chao 1 indices for river water samples were both higher than those for co-cultivated samples. This result contradicts previous findings showing that both the evenness and richness of microbes inhabiting natural duckweeds were higher than those found in environmental waters (Acosta *et al.*, 2020; Iwashita *et al.*, 2020; Bunyoo *et al.*, 2022). This may be due to duckweeds in the present study being grown in a so-called closed system, in which no new microbial source was provided from the outside after the initial inoculation with the microbial community, while the previous study targeted duckweeds grown in a natural environment in which different microbial communities are constantly provided.

**Fig. 2.**
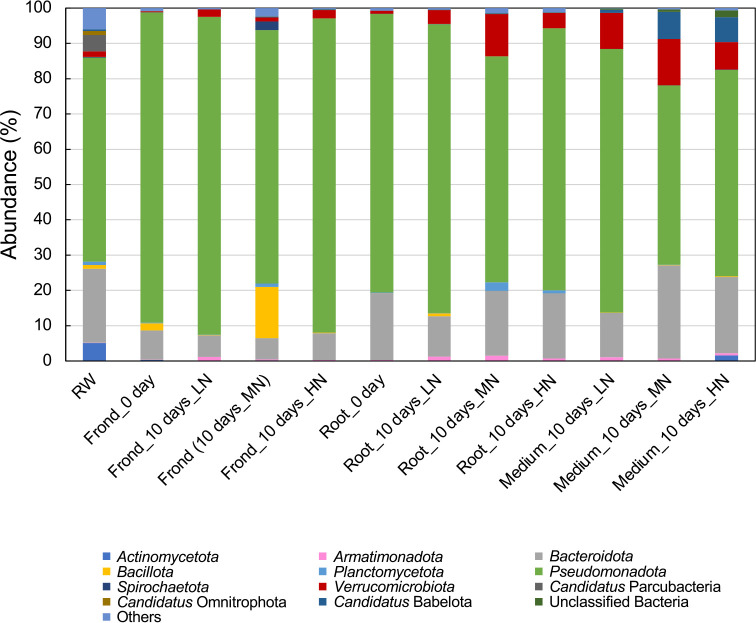
Microbial compositions in samples from the “duckweed-microbes co-cultivation system” at the phylum level. Sequences that were not classified into particular groups and those of the taxa indicating their maximum abundance <1.0% in each sample were assembled as “Others”.

As shown in Fig. 2, *Pseudomonadota* was the dominant phylum in all samples with a rate of 51–90%, followed by‍ ‍the phylum *Bacteroidota*, except for the fronds of MN (middle concentration of nitrogen) medium. Regarding other phyla, the abundance of *Verrucomicrobiota*, which is a rarely cultivated bacterial phylum, was slightly higher in duckweed roots and co-cultivated medium samples recovered from all three culture bottles including LN (low concentration of nitrogen), MN, and HN (high concentration of nitrogen) medium after 10 days of co-cultivation than in river water samples (1.5%), the microbial inoculum source. Distribution rates were 4.0% (LN medium; Root_10 days_LN), 12.0% (MN medium; Root_10 days_MN), and 4.4% (HN medium; Root_10 days_HN) for the roots, and 10.2% (LN medium; Medium_10 days_LN), 13.0% (MN medium; Medium_10 days_MN), and 7.8% (HN medium; Medium_10 days_HN) for co-cultivated media. In contrast, the distribution rates of *Verrucomicrobiota* did not markedly differ between frond samples and river water samples after co-cultivation, but exceeded 1% in all experimental sections (2.0% for LN medium (Frond_10 days_LN), 1.1% for MN medium (Frond_10 days_MN), and 2.4% for HN medium (Frond_10 days_HN)).

In comparisons of the distribution rates of *Verrucomicrobiota* in the roots and fronds (0.9 and 0.3%, respectively) immediately after the inoculation (day 0) with the microbes in river water samples, scores increased by 4.9- (LN medium), 13.3- (MN medium), and 4.4-fold (HN medium) in the roots and by 6.7- (LN medium), 3.7- (MN medium), and 8.0-fold (HN medium) in the fronds after 10‍ ‍days of co-cultivation, suggesting that the microbes of this phylum exhibited dominant growth over other bacterial species at each site after attaching to the plant. The high frequency of the strains belonging to the phylum *Verrucomicrobiota* obtained from the roots of duckweed in our previous study may be attributed to their propensity to dominate in the roots.

**Fig. 3.**
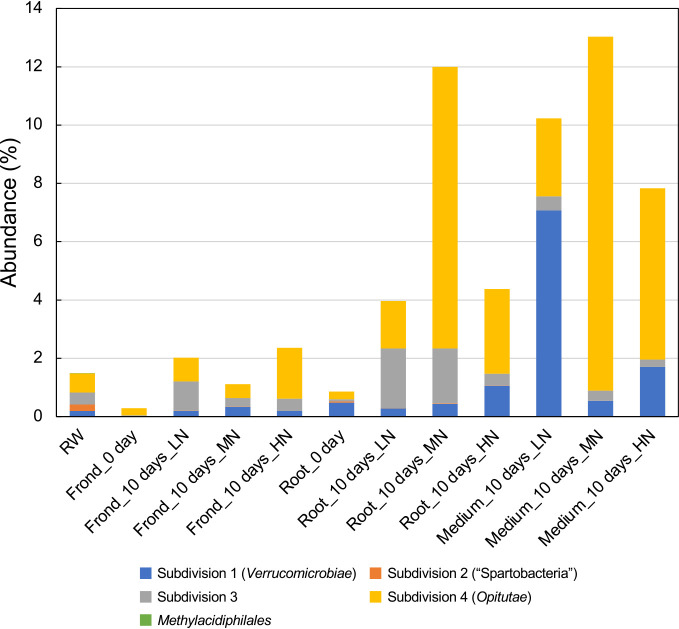
Composition of *Verrucomicrobiota* detected in samples from the “duckweed-microbes co-cultivation system” at the subdivision level.

Previous studies that focused on aquatic environmental samples showed that the abundance of *Verrucomicrobiota* was affected by total nitrogen, nitrate, or ammonium concentrations (Haukka *et al.*, 2006; Chiang *et al.*, 2018; Pei *et al.*, 2021); however, this phenomenon was not observed in our co-cultivation system. Nevertheless, when examining the constituent members of *Verrucomicrobiota* at the subdivision level in each sample, we found that subdivision 4 (*Opitutae*) was the major component in the roots, fronds, and co-cultivated medium in the co-culture system using MN and HN media. In contrast, subdivision 3 was the major component in the roots and fronds in the system using LN medium, while subdivision 1 (*Verrucomicrobiae*) was the major component in the co-cultivated medium in the system using LN medium (Fig. 3). Shen *et al.* (2017) previously reported that constituent members of the phylum *Verrucomicrobiota* in soil slightly varied with the concentration of nitrogen. Their findings indicated a negative correlation between the microbes of subdivision 3 and total nitrogen, which is consistent with the present results. However, the relationship between subdivisions 1 and 4 was opposite to that in the present study; subdivision 1 positively correlated and subdivision 4 negatively correlated with total nitrogen. While this difference may be due to variations in soil and aquatic environments, further studies targeting a larger number of samples are needed to clarify the underlying reasons.

**Fig. 4.**
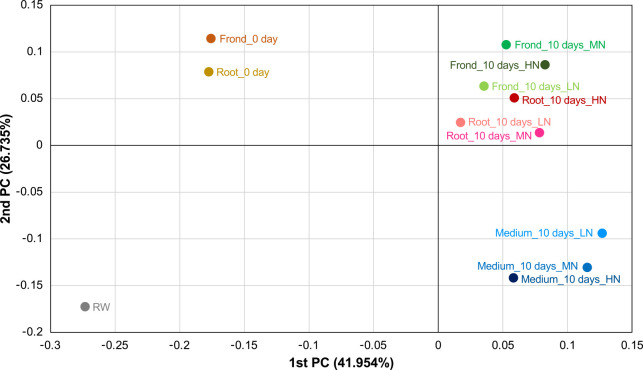
Principal coordinates ana­lysis of microbial communities using the Jansen-Shannon metric. Data at the genus level that exclude unclassified OTUs were used.

To perform a more detailed comparison of microbial communities in each sample, we exami­ned results at the genus level (Fig. S2). Eighty-three bacterial genera, the abundance of which was >1% in at least one sample, were detected. Using these data, PCoA and hierarchical cluster heat map ana­lyses were conducted. As shown in Fig. 4, the microbial communities in river water and plant samples before the co-cultivation markedly differed from those in samples after the co-cultivation. In addition, samples after the co-cultivation were further classified into two major groups, water samples and plant-related samples. Similar results emerged in the hierarchical cluster heat map, showing that the microbial composition in samples from the co-cultivation systems using MN and HN media were more similar for each of the fronds, roots, and co-cultivated medium than those in the system using LN medium (Fig. S3). This similarity may be attributed not only to the direct effects of the different medium compositions on the microbial community, but also to the nitrogen content of LN medium being sufficient for the growth of duckweed (Malla *et al.*, 2010). The growth of aseptic duckweed in LN medium was retarded with the formation of turions, and some of their fronds turned yellowish after 10 days of cultivation (data not shown); therefore, metabolism in the plant (*i.e.*, the quantity and quality of exudates from the roots and fronds) may have differed from that of the two other media.

We previously reported marked differences in the microbial communities inhabiting the roots and fronds of duckweed (Iwashita *et al.*, 2020). In the present study, similar results were observed in the experimental systems on MN and HN media (Fig. S3). However, in the LN medium system, the microbial community structure between the roots and fronds showed fewer variations than those in the two other experimental systems (Fig. S3). On the other hand, some bacterial groups specifically dominated in frond (*e.g.*, *Niveispirillum*, *Pseudacidovorax*, and *Sphingomonas*) or root samples (*e.g.*, *Haliscomenobacter*, unclassified Saprospiraceae, and Unclassified Thiobacillaceae) across all‍ ‍co-cultivation systems (Table S3). These results clearly indicated that not only the roots, but also the fronds and co-cultivated medium need to be used for microbial isolation in the duckweed-microbes co-cultivation system, particularly those using MN and HN media. Furthermore, by adjusting cultivation conditions, such as nitrogen concentrations in the culture medium, it may be possible to obtain a larger variety of isolates from a single environmental sample (microbial inoculum).

### Isolation of *Verrucomicrobiota* from duckweed-microbes co-cultivation systems

The results of the culture-independent ana­lysis showed that *Verrucomicrobiota* members became dominant in the roots and co-cultivated medium of the co-cultivation systems. We previously revealed that the roots of duckweed after a co-cultivation were an effective source for isolating taxonomically novel microbes, including this phylum (Tanaka *et al.*, 2018). However, the general potential of the fronds and the co-cultivated medium as sources of microbial isolation had not been exami­ned. To address this, we isolated 490 strains from root, frond, and co-cultivated medium samples after co-cultivation (Table S2), and subjected them to a screening system using PCR primers that specifically detect *Verrucomicrobiota* (no isolate was obtained from the fronds of the HN medium system due to swarming bacteria covering the surface on the plate during the culture process). Screening identified 44 isolates of *Verrucomicrobiota*, all from samples related to the co-cultivation systems (490 strains), while none of the 50 strains from river water passed the screening (Table S2).

Among *Verrucomicrobiota* isolates, 32 strains were from co-cultivated media (18 from LN medium, 11 from MN medium, and 3 from HN medium), which showed high populations of the phylum in the culture-independent ana­lysis. In addition, the bacterial group was isolated from the roots that showed an increased abundance of this phylum as well as from co-cultivated media, but not from all samples; two strains were from each of the samples in MN and HN media, while no isolate was obtained from the sample in LN‍ ‍medium. On the other hand, the abundance of *Verrucomicrobiota* was lower in frond samples than in root and co-cultivated medium samples and was similar to that in river water samples in the culture-independent ana­lysis; however, one and seven strains of this phylum were isolated from the fronds in MN and LN media, respectively. These results suggest that the ease of isolating the rarely cultivated bacterial group from duckweed-related samples is attributed to their dominance in the samples during co-cultivation (particularly the roots and cultivating medium). Additionally, the effects of various factors from the plants may contribute to changing the microbes into a cultivable state.

Comparisons of 16S rRNA gene sequences from *Verrucomicrobiota* isolates with sequences in the NCBI database revealed the taxonomic positions of 39 out of 44 strains; 18 and 21 strains were members of subdivision 1 (*Verrucomicrobiae*) and subdivision 4 (*Opitutae*), respectively (Table 1). Regarding the remaining 5 strains (AR-F44LN, AR-F46LN, AR-F58LN, AR-F26MN, and AR-R30HN), se­quence similarities to those of authentic *Verrucomicrobiota* species were low, preventing a definitive taxonomic assignment. Therefore, we constructed a phylogenetic tree of these strains and their relatives, and found that all five strains were members of subdivision 3 (Fig. S4). These results mostly reflect data obtained through a culture-independent microbial community ana­lysis. For example, as shown in Fig. 3, *Verrucomicrobiae* and *Opitutae* were the dominant group of the phylum detected in LN and MN media, respectively, and the majority of isolates from each sample (17 of 18 isolates for LN medium and 10 of 11 isolates for MN medium) also fell into their corresponding taxa.

Chemical and physical factors, such as nutritional conditions, temperature, and light intensity, affect the composition of root exudates in plants (Printon *et al.*, 2007). Therefore, the present results suggest that changing the taxonomic composition of *Verrucomicrobiota* in the co-cultivation system by manipulating factors that affect the growth and/or metabolism of duckweed, such as nitrate concentrations in the medium, may lead to the development of an enhanced duckweed-microbes co-cultivation method. While the targets are limited to several members of *Verrucomicrobiota* adaptable to co-cultivation with duckweed, this improved method has the potential to facilitate the isolation of a wider range of microbes within this phylum from a single environmental sample, complementing the efficacy of the current method. We are actively investigating physical and chemical factors that may efficiently change the composition of members within the phylum *Verrucomicrobiota* in the co-cultivation system.

## Citation

Tanaka, Y., Tozawa, E., Iwashita, T., Morishita, Y., Tamaki, H., Toyama, T., et al. (2024) Successful Isolation of Diverse *Verrucomicrobiota* Strains through the Duckweed-Microbes Co-cultivation Method. *Microbes Environ ***39**: ME24019.

https://doi.org/10.1264/jsme2.ME24019

## Supplementary Material

Supplementary Material

## Figures and Tables

**Table 1.** *Verrucomicrobiota* isolates and their related authentic species based on the 16S rRNA gene sequence

Sample	Strain	Subdivision	Closest authentic species (Accession No.)	Identity (%)	Compared Length (bp)
Frond_10 days_LN	AR-F20LN	4	*Horticoccus luteus* (CP080507)	93.95	857
	AR-F22LN	4	*Lacunisphaera limnophila* (NR_146349)	97.24	833
	AR-F36LN	4	*Horticoccus luteus* (CP080507)	93.59	840
	AR-F44LN	3	*Fontisphaera persica* (CP116615)	86.73	844
	AR-F46LN	3	*Fontisphaera persica* (CP116615)	86.73	867
	AR-F50LN	4	*Lacunisphaera limnophila* (NR_146349)	97.49	837
	AR-F58LN	3	*Fontisphaera persica* (CP116615)	86.76	777
Frond_10 days_MN	AR-F26MN	3	*Fontisphaera persica* (CP116615)	86.61	854
Root_10 days_MN	AR-R66MN	4	*Oleiharenicola lentus* (NR_170425)	98.84	862
	AR-R75MN	4	*Oleiharenicola lentus* (NR_170425)	99.50	795
Root_10 days_HN	AR-R30HN	3	*Fontisphaera persica* (CP116615)	86.71	860
	AR-R57HN	4	*Oleiharenicola lentus* (NR_170425)	96.55	867
Medium_10 days_LN	AR-M6LN	1	*Luteolibacter flavescens* (NR_156157)	94.76	818
	AR-M14LN	1	*Luteolibacter flavescens* (NR_156157)	94.70	793
	AR-M34LN	4	*Horticoccus luteus* (CP080507)	94.02	867
	AR-M44LN	1	*Luteolibacter flavescens* (NR_156157)	94.92	844
	AR-M47LN	1	*Luteolibacter flavescens* (NR_156157)	94.74	817
	AR-M48LN	1	*Luteolibacter flavescens* (NR_156157)	94.88	820
	AR-M49LN	1	*Luteolibacter flavescens* (NR_156157)	95.27	698
	AR-M50LN	1	*Luteolibacter flavescens* (NR_156157)	94.77	840
	AR-M61LN	1	*Luteolibacter flavescens* (NR_156157)	94.76	818
	AR-M63LN	1	*Luteolibacter flavescens* (NR_156157)	94.91	824
	AR-M68LN	1	*Luteolibacter flavescens* (NR_156157)	94.92	827
	AR-M70LN	1	*Luteolibacter flavescens* (NR_156157)	95.06	852
	AR-M71LN	1	*Luteolibacter flavescens* (NR_156157)	94.84	813
	AR-M74LN	1	*Luteolibacter flavescens* (NR_156157)	94.76	818
	AR-M78LN	1	*Luteolibacter flavescens* (NR_156157)	94.91	867
	AR-M81LN	1	*Luteolibacter flavescens* (NR_156157)	94.95	852
	AR-M86LN	1	*Luteolibacter flavescens* (NR_156157)	95.06	849
	AR-M94LN	1	*Luteolibacter flavescens* (NR_156157)	94.77	845
Medium_10 days_MN	AR-M9MN	4	*Horticoccus luteus* (CP080507)	91.91	863
	AR-M25MN	4	*Oleiharenicola alkalitolerans* (KJ721192)	92.77	838
	AR-M27MN	4	*Horticoccus luteus* (CP080507)	92.08	871
	AR-M29MN	4	*Horticoccus luteus* (CP080507)	91.97	856
	AR-M36MN	4	*Oleiharenicola alkalitolerans* (KJ721192)	92.84	839
	AR-M37MN	4	*Horticoccus luteus* (CP080507)	93.47	853
	AR-M46MN	4	*Horticoccus luteus* (CP080507)	92.56	856
	AR-M47MN	4	*Oleiharenicola alkalitolerans* (KJ721192)	92.84	846
	AR-M50MN*	1	*Luteolibacter ambystomatis* (CP073100)	97.46	790
	AR-M65MN	4	*Horticoccus luteus* (CP080507)	92.00	859
	AR-M76MN	4	*Horticoccus luteus* (CP080507)	91.90	838
Medium_10 days_HN	AR-M54HN	4	*Horticoccus luteus* (CP080507)	91.94	866
	AR-M60HN	4	*Horticoccus luteus* (CP080507)	92.00	860
	AR-M89HN	4	*Oleiharenicola alkalitolerans* (KJ721192)	92.84	849

* The 16S rRNA gene of this strain was sequenced with the primer EUB-1500R
