# Supplementary material for: Successful Isolation of Diverse Verrucomicrobiota Strains through the Duckweed-Microbes Co-cultivation Method
Source: Microbes Environ. 2024 Sep 12;39(3):ME24019. doi: 10.1264/jsme2.ME24019 (PMC11427312; doi:10.1264/jsme2.ME24019)
Supplement: Supplementary file 1 — Supplementary Material [file 39_24019_s1.pdf]

Supplementary Materials

Successful Isolation of Diverse *Verrucomicrobiota* Strains through the Duckweed-Microbes Co-cultivation Method

YASUHIRO TANAKA, ERINA TOZAWA, TOMOKI IWASHITA, YOSUKE MORISHITA, HIDEYUKI TAMAKI, TADASHI TOYAMA, MASAAKI MORIKAWA, YOICHI KAMAGATA and KAZUHIRO MORI

Table S1. Number of reads, OTUs, and alpha diversity indices for microbial communities in samples

Sample	Target reads	OTUs	Chao 1 (Richness)	Shannon (Evenness)	Good's coverage of library(%)
RW	91578	7734	7888.58	6.77	99.04
Frond_0 day	4449	343	404.56	4.35	97.89
Frond_10 days_LN)	48688	663	739.68	4.48	99.74
Frond_10 days_MN	26145	689	739.38	4.81	99.58
Frond_10 days_HN	19997	435	469.67	4.23	99.67
Root_0 day	31407	915	963.65	4.48	99.55
Root_10 days_LN	92280	916	1000.30	4.38	99.83
Root_10 days_MN	60914	1033	1099.92	4.98	99.75
Root_10 days_HN	66981	943	996.55	4.77	99.8
Medium_10 days_LN	97209	682	711.26	4.18	99.92
Medium_10 days_MN	90190	808	849.53	4.22	99.88
Medium_10 days_HN	89915	662	694.41	3.97	99.91

Table S2. Number of *Verrucomicrobiota* isolates obtained in this study

Sample	Screened strains	*Verrucomicrobiota* strains	Yield (%)
RW	50	0	0
Frond_10 days_LN	68	7	10.3
Frond_10 days_MN	72	1	1.4
Frond_10 days_HN*	–	–	–
Root_10 days_LN	29	0	0
Root_10 days_MN	60	2	3.3
Root_10 days_HN	41	2	4.9
Medium_10 days_LN	81	18	22.2
Medium_10 days_MN	69	11	15.9
Medium_10 days_HN	70	3	4.3

*Due to the swarming behavior of bacteria growing on the microbial isolation plate, no isolate was obtained.

Table S3. Microbial abundance in duckweed fronds and roots at the genus level after co-cultivation

Taxon Name	Frond_10 days_LN	Frond_10 days_MN	Frond_10 days_HN	Root_10 days_LN	Root_10 days_MN	Root_10 days_HN
Acidovorax	**3.108**	**2.215**	**2.530**	**4.107**	**1.850**	**4.225**
Albidiferax	**1.415**	**1.434**	**1.255**	**3.575**	**1.481**	**1.351**
Aquabacterium	0.965	**1.702**	0.730	0.249	0.067	0.082
Aquincola	**1.534**	**1.212**	0.180	**1.604**	0.314	0.211
Asprobacter	0.854	0.157	0.135	**1.104**	**1.169**	**1.000**
Asticcacaulis	**5.617**	**2.000**	**2.785**	**3.300**	0.831	1.118
Azohydromonas	**1.148**	0.268	0.295	**1.659**	**2.609**	**2.874**
Caulobacter	0.883	**1.109**	**2.255**	0.757	0.561	0.867
Clostridium	0.000	**9.551**	0.000	0.134	0.000	0.000
Comamonas	**8.351**	0.956	**1.260**	**14.368**	**4.408**	**3.301**
Delftia	**4.204**	**1.805**	**1.060**	**4.984**	**2.139**	**1.756**
Diplosphaera	0.335	0.249	0.955	**1.044**	**4.240**	**1.227**
Emticicia	0.277	0.176	0.945	0.094	0.653	**1.251**
Flavobacterium	0.774	0.122	0.190	**4.193**	0.389	0.346
Flectobacillus	0.598	0.994	0.980	0.270	**3.812**	0.576
Georgfuchsia	0.641	0.180	0.070	**1.337**	0.131	0.057
Haliscomenobacter	0.004	0.061	0.175	0.151	**1.458**	**4.640**
Hydrogenophaga	**1.366**	**1.362**	**1.640**	0.968	0.629	**2.390**
Inhella	0.571	**1.147**	0.985	0.491	0.200	0.764
Lacihabitans	0.148	0.528	**1.195**	0.191	0.924	**2.213**
Leptothrix	**2.237**	0.830	0.400	0.626	0.284	0.591
Limnobacter	0.986	**1.362**	**9.491**	0.041	**1.656**	0.642
Methylibium	0.877	0.589	0.320	**1.391**	**1.159**	**1.771**
Methylophilus	**3.867**	**6.732**	**13.322**	**1.428**	**2.233**	**3.065**
Methylotenera	0.516	**3.308**	**1.175**	0.627	**1.379**	0.761
Niveispirillum	**1.142**	**1.235**	**1.670**	0.232	0.172	0.069
Novosphingobium	**5.443**	**2.643**	**2.245**	**3.067**	**1.931**	**1.962**
Opitutus	0.361	0.229	0.600	0.468	**4.285**	**1.408**
Parasediminibacterium	0.454	0.210	0.840	0.841	**1.095**	**1.500**
Pelomonas	**4.143**	**3.117**	**2.305**	**7.752**	**2.065**	**2.013**
Phenylobacterium	**1.545**	**1.159**	0.240	0.677	0.314	0.148
Phreatobacter	0.000	**1.040**	0.305	0.000	0.011	0.284
Pseudacidovorax	0.099	**3.140**	**9.056**	0.013	0.289	0.127
Rectinema	0.000	**2.410**	0.000	0.036	0.000	0.000
Rhizobium	0.861	0.715	**2.560**	0.540	0.781	**1.596**
Rhodobacter	**3.116**	0.413	0.720	**1.661**	**1.937**	**3.459**
Rhodoferax	**1.089**	0.340	0.585	0.743	0.128	0.273
Rubrivivax	**2.173**	**1.063**	0.830	0.377	0.284	0.285
Sediminibacterium	0.201	0.524	0.815	0.056	0.284	**1.094**
Sphaerotilus	0.655	0.421	**1.815**	0.200	0.154	**1.692**
Sphingomonas	**1.524**	**1.090**	**3.265**	0.050	0.039	0.336
Sphingorhabdus	0.700	0.949	**1.655**	0.708	**2.182**	**3.289**
Trichococcus	0.000	**1.698**	0.000	0.548	0.000	0.000
Unclassified Brucellaceae	**2.113**	0.130	0.130	0.386	0.039	0.013
Unclassified Comamonadaceae 1	**6.761**	**1.717**	**1.705**	**5.219**	**1.655**	**2.451**
Unclassified Comamonadaceae 3	**4.412**	**2.119**	**1.155**	**2.747**	**1.244**	**1.365**
Unclassified Comamonadaceae 4	**1.284**	0.574	0.520	0.657	0.629	0.775
Unclassified Flavobacteriales	0.440	0.566	0.905	0.829	**1.024**	0.688
Unclassified Methylophilaceae	**1.273**	**8.189**	**6.221**	**2.069**	**8.489**	**7.996**
Unclassified Opitutaceae	0.113	0.008	0.185	0.106	**1.098**	0.273
Unclassified Pedosphaerae	0.994	0.122	0.425	**1.944**	**1.413**	0.311
Unclassified Planctomycetaceae	0.101	0.731	0.025	0.106	**1.955**	0.487
Unclassified Saprospiraceae	0.014	0.149	0.050	0.043	**1.788**	**1.003**
Unclassified Thiobacillaceae	0.064	0.080	0.030	0.230	**1.139**	0.430

Only genera with abundance >1% (bold) in at least 1 sample were shown.

Pale green: more than 2-fold the score at the root; Green: more than 5-fold the score at the root; Pale blue: more than 2-fold the score at the frond; Blue: more than 5-fold the score at the frond

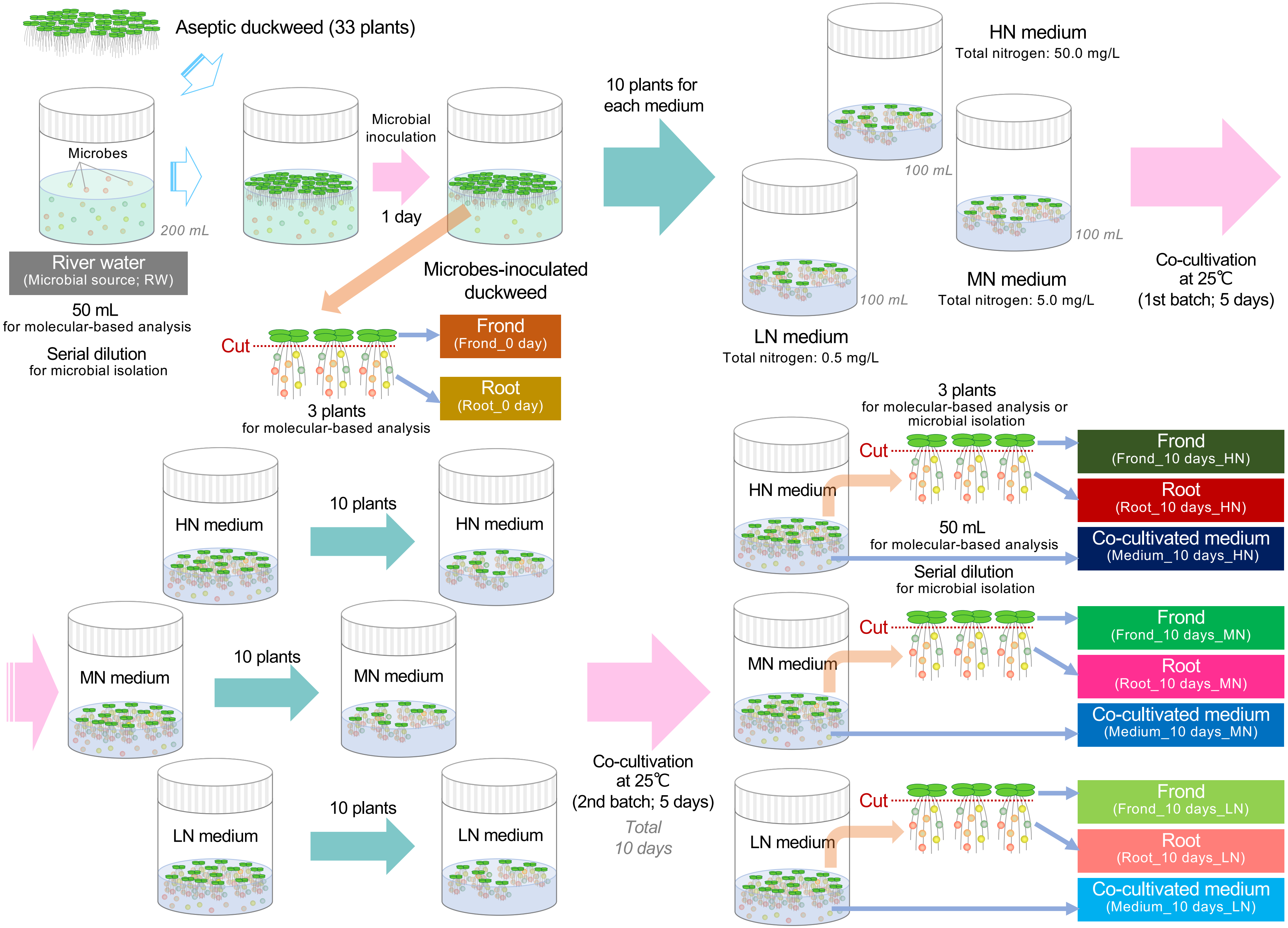

Fig. S1. Schematic image of the microbes-duckweed co-cultivation systems used in this study

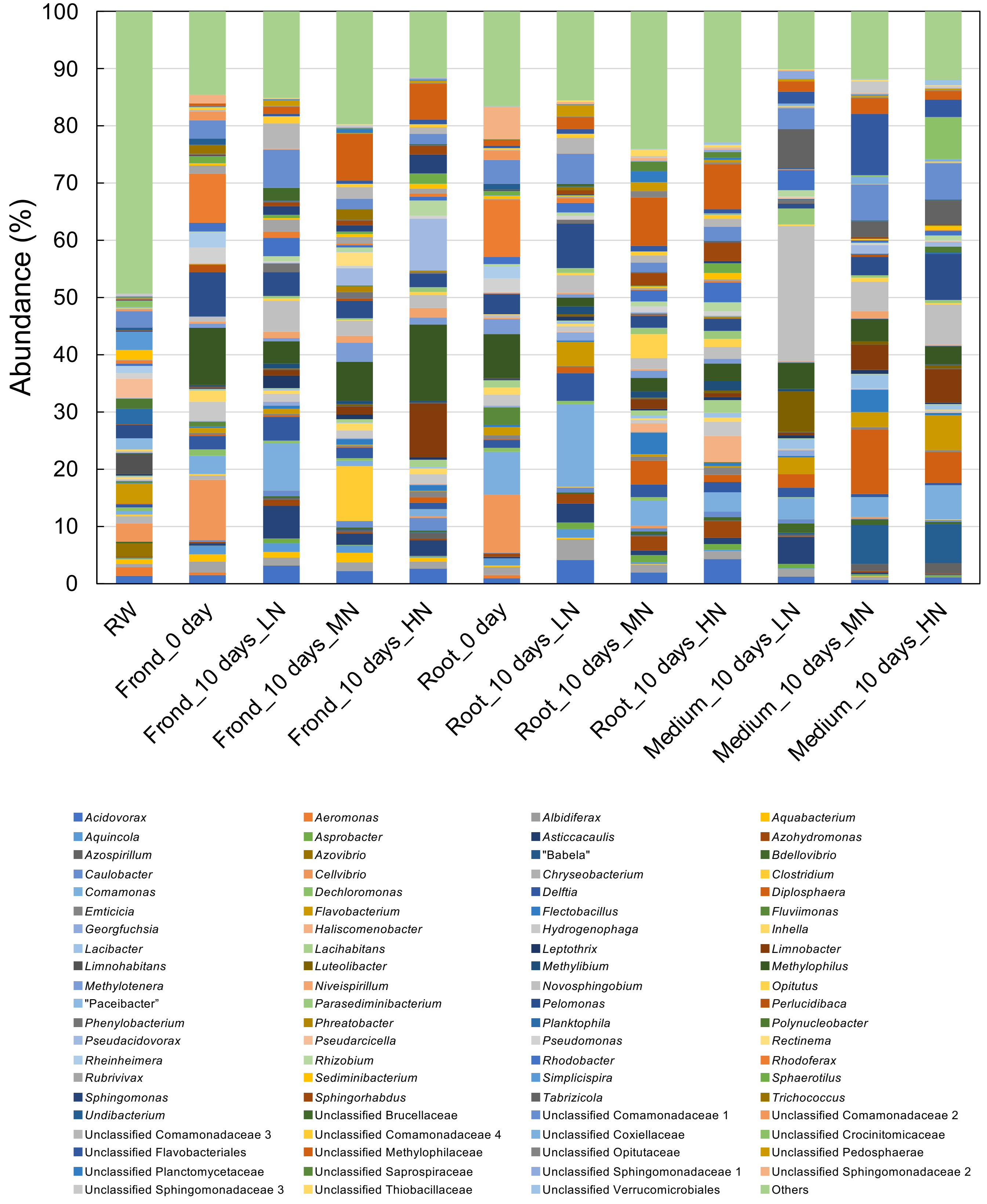

Fig. S2. Microbial compositions in the samples from the “duckweed-microbes co-cultivation system” at the genus level. Sequences that were not classified into particular groups and those of taxa with a maximum abundance <1.0% in each sample were assembled as “Others”.

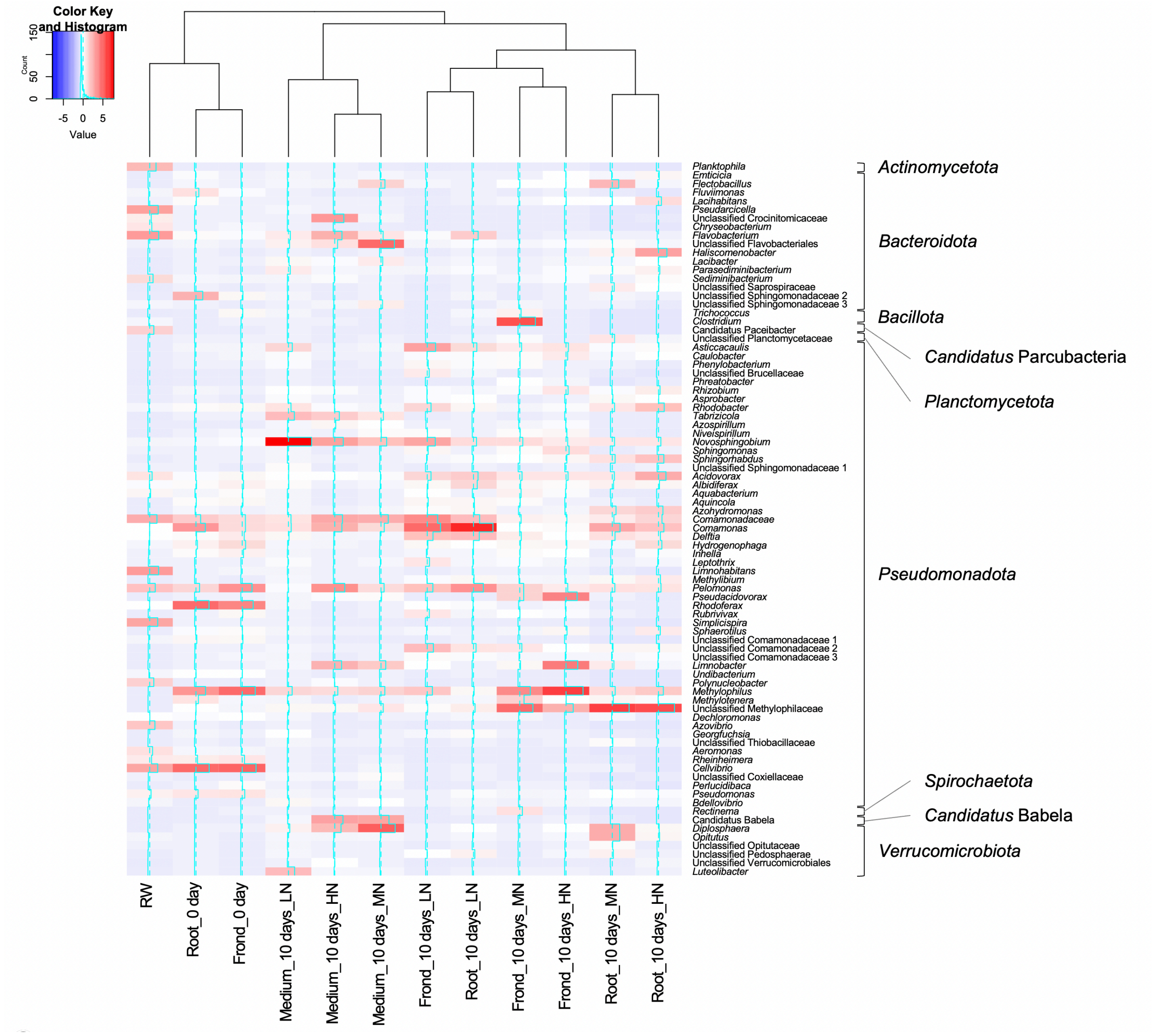

Fig. S3. Heat map for the distribution of bacterial genera in the samples from the "duckweed-microbes co-cultivation system". Taxa with a maximum abundance <1.0% in each sample were not included.

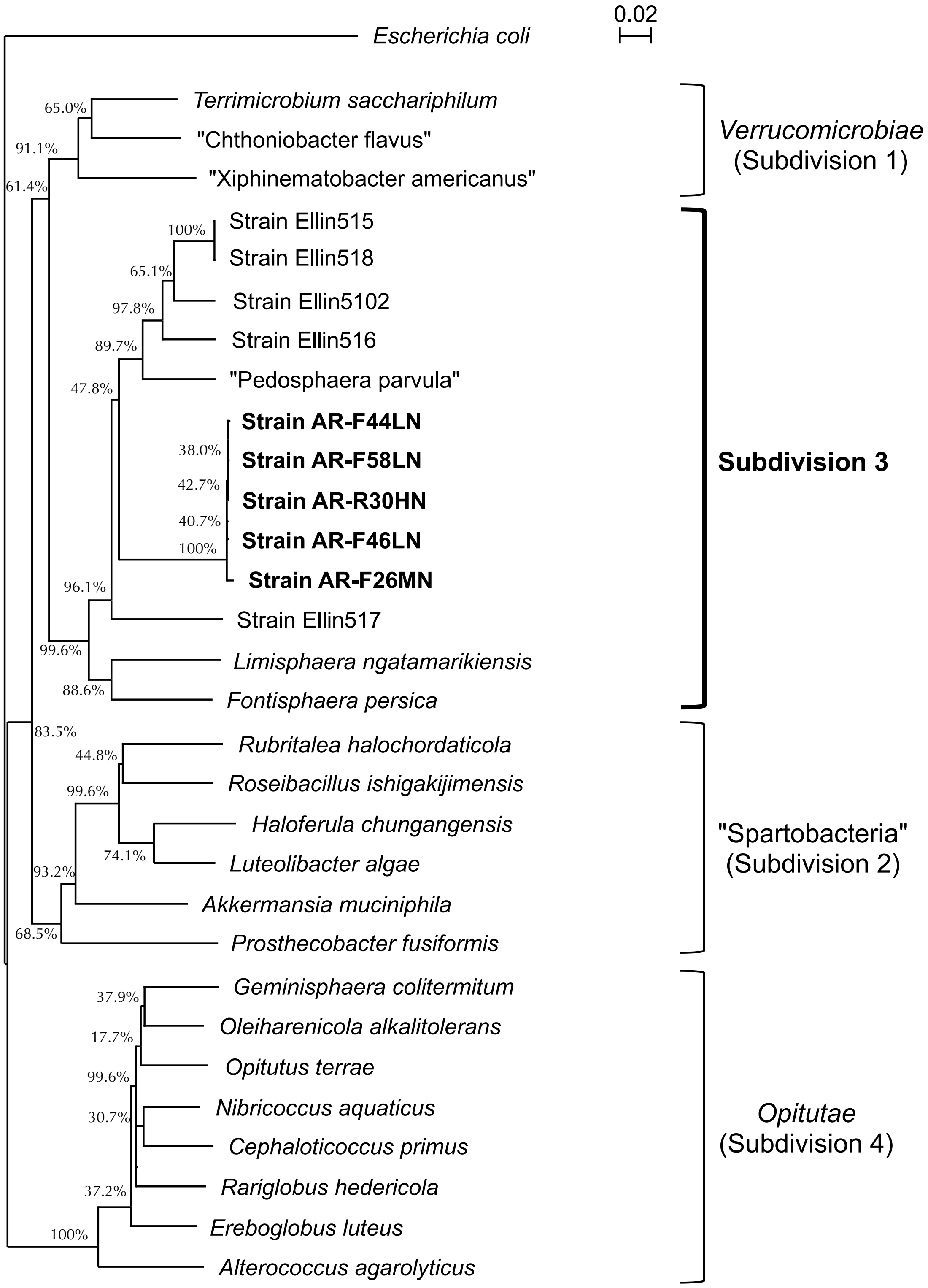

Fig. S4. Phylogenetic tree showing the relationships between the strains AR-F44LN, AR-F46LN, AR-F58LN, AR-F26MN and AR-R30HN, and their related sequences of *Verrucomicrobiota*. The tree was constructed using the neighbor-joining (NJ) method based on the 16S rRNA gene sequences. The percentages shown in nodes were bootstrap values estimated using the NJ method. The scale bar indicates 0.02 substitutions per nucleotide position.
